# Transcriptome Analyses Show Changes in Gene Expression Triggered by a 31-bp InDel within *OsSUT3* 5′UTR in Rice Panicle

**DOI:** 10.3390/ijms241310640

**Published:** 2023-06-26

**Authors:** Qiuping Li, Chunlong Zhang, Jiancheng Wen, Lijuan Chen, Yitong Shi, Qinghui Yang, Dandan Li

**Affiliations:** 1 Rice Research Institute, Yunnan Agricultural University, Kunming 650201, China; qiupingyouxiang@163.com (Q.L.); zhangchl1013@163.com (C.Z.); jcwen1117@163.com (J.W.); 1986014@ynau.edu.cn (L.C.); 2 College of Agronomy and Biotechnology, Yunnan Agricultural University, Kunming 650201, China; m13988499100@163.com

**Keywords:** transcriptome, InDel, sucrose, pollen fertility, rice

## Abstract

Pollen development and its fertility are obligatory conditions for the reproductive success of flowing plants. *Sucrose transporter 3* (*OsSUT3*) is known to be preferentially expressed and may play critical role in developing pollen. A 31-bp InDel was identified as a unique variation and was shown to be responsible for the expression of downstream gene in our previous study. In this study, to analyze the changes of gene expression triggered by 31-bp InDel during pollen development, two vectors (*p385-In/Del::OsSUT3-GUS*) were constructed and then stably introduced into rice. Histochemical and quantitative real-time PCR (qRT-PCR) analysis of transgenic plants showed that 31-bp deletion drastically reduced the expressions of downstream genes, including both *OsSUT3* and *GUS* in rice panicle at booting stage, especially that of *OsSUT3*. The transcriptome profile of two types of panicles at booting stage revealed a total of 1028 differentially expressed genes (DEGs) between 31-bp In and 31-bp Del transgenic plants. Further analyses showed that 397 of these genes were significantly enriched for the ‘metabolic process’ and ‘binding’. Among them, nineteen genes had a strong relationship with starch and sucrose metabolism and were identified as candidate genes potentially associated with the starch accumulation in rice pollen, which that was also verified via qRT-PCR. In summary, 31-bp InDel plays a crucial role not only in the regulation of downstream genes but in the expression of sucrose-starch metabolizing genes in multiple biological pathways, and provides a different regulation mechanism for sucrose metabolism in pollen.

## 1. Introduction

Rice (*Oryza sativa* L.) is the world’s major food crop. Sucrose, as a precursor substrate for starch synthesis, is essential to the yield and quality of rice. In plants with higher sucrose values, nearly 80% of photosynthetic products are transported from the source to sink organs mainly in the form of sucrose [1]. Sucrose transporters (SUTs) have been demonstrated to play significant parts in the transmembrane transport of sucrose and its distribution in various plants [2,3]. Before sucrose is transported to the phloem, the short distance loading of sucrose and the regulation of external environment adaptation, such as temperature and photoperiod, both depend on SUTs [4].

In rice, there are five *SUT* genes identified, including *OsSUT1-5* [5]. As an important member of the *OsSUTs* gene family, *OsSUT3* is preferentially expressed in rice pollen by a pollen-specific promoter [6,7,8,9]. A 31-bp deletion located in 5′UTR of *OsSUT3* obviously reduced rice seeding rate and fertile pollen number probably due to the decreased expression of the gene [10]. However, except for the *OsSUT3* gene, comprehensive expression analysis of related genes is necessary to measure the contribution of 31-bp InDel to pollen development in rice.

It is well-known that insertions–deletions (InDels) of nucleotide sequences in different regions is an important phenomenon during biological evolution [11]. Upstream noncoding regions, such as promoter and 5′UTR, contain a number of cis-acting elements responsive to the various expression pattern of gene and the environmental changes [12]. For the *FT* gene, which regulates the flowering time in a range of plant families, a staggering number of InDels within the promoter ranging in size from 20 to 6850 bp have been identified in wild and domestic wheats [13,14,15,16]. Similarly, a 598-bp InDel variation in the promoter region of *Bna.SOC1.A05* has a huge impact on mRNA expression and is associated with winter types in rapeseeds [17]. Both in plants and animals, InDel mutations were highly enriched in the driving region of gene and associated with differential gene expression and changes [18,19].

DEGs can be easily detected among accessions using transcriptome sequencing and genetic analysis, and they may be one of the most abundant clues to identify the molecular regulation mechanism of phenotypic change in plant [20]. To investigate the differences of gene expression patterns between the 31-bp In and Del transgenic rice plants at the late stage of pollen development, the transcriptome sequencing of panicle and qRT-PCR were performed in our study. Base on a detailed data analysis, many changes in gene expression associated with pollen development were identified. Additionally, it is important to understand the effects of 31-bp InDel on the expression and function of pollen-development-related genes.

## 2. Results

### 2.1. Improvement of Pollen and Panicle-Related Traits in 31-bp in Transgenic Lines

**Figure 1:**
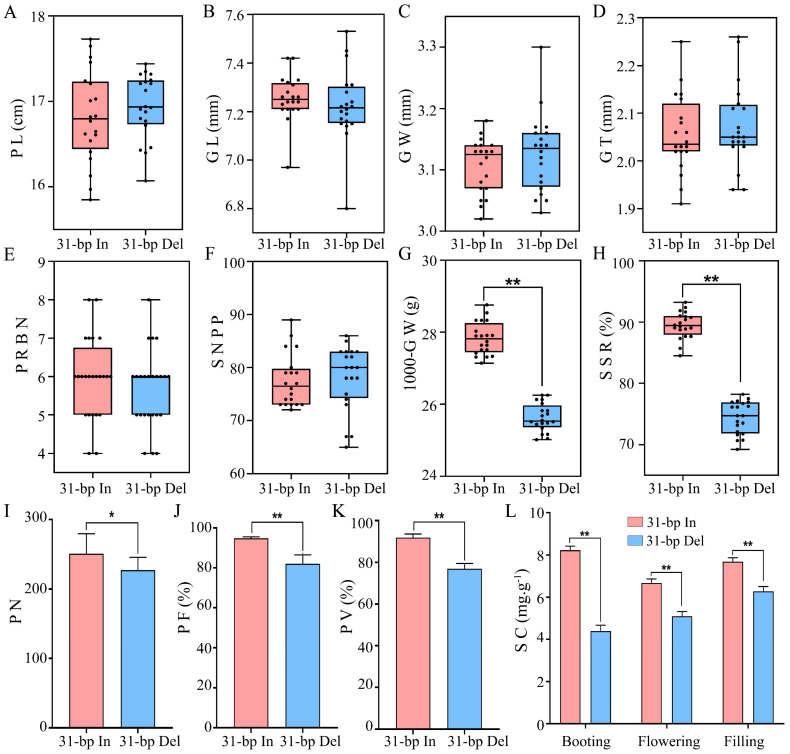
Comparative analysis of panicle-related traits and pollen fertility. (**A**): panicle length; (**B**): grain length; (**C**): grain width; (**D**): grain thickness; (**E**): primary rachis branch number; (**F**): spikelet number per panicle; (**G**): 1000-grain weight; (**H**): seed setting rate; (**I**): pollen number; (**J**): pollen fertility; (**K**): pollen viability; (**L**): sucrose content (** *p* < 0.01, * *p* < 0.05, the significance of the difference was analyzed using *t*-test).

We previously evaluated architecture-related morphological traits in 560 rice accessions including 513 varieties with 31-bp insertion and 47 varieties where the 31-bp had been deleted. Phenotypic analyses showed that three panicle- and pollen-related traits, including seed setting rate (SSR), panicle length (PL), and fertile pollen number (FPN), increased significantly in rice accessions with 31-bp [10]. In this study, we performed significant analysis using P-values of the eight panicle-related traits and three pollen-related traits, including PL, primary rachis branch number (PRBN), grain length (GL), grain width (GW), grain thickness (GT), 1000-grain weight (1000-GW), spikelet number per panicle (SNPP), SSR, pollen number (PN), pollen fertility (PF), and pollen viability (PV) (Figure 1A). Among these traits, we found that 1000-GW and SSR, which best reflects the grain yield per plant (GYPP), increased significantly in rice accessions with 31-bp (*p* < 0.01), 16.87% and 7.96%, respectively (Figure 1G,H and Figure S1A). PF and PV, which are critical for yielding fully fertile seeds, were significantly higher in rice varieties including 31-bp insertion fragments, 15.48% and 19.38%, respectively (*p* < 0.01) (Figure 1J,K and Figure S1B,C). These results indicated that 31-bp insertion in 5′UTR of *OsSUT3* may contribute to the increase in GYPP including panicle- and pollen-related traits during rice breeding.

Additionally, the sucrose content (SC) in panicle affecting pollen fertility and GYPP showed a significant difference (*p* < 0.01) between 31-bp In and Del transgenic plants at three stages of late pollen development (Figure 1L). SC significantly decreased by 46.64%, 23.72% and 18.24% due to 31-bp deletion at booting, flowering, and filling stages, respectively. Therefore, we deduced that 31-bp sequence likely plays a role in regulating starch accumulation during pollen development.

### 2.2. Expression Changes in OsSUT3 and GUS in Different Tissues and Different Developmental Stage of Transgenic Plants

**Figure 2:**
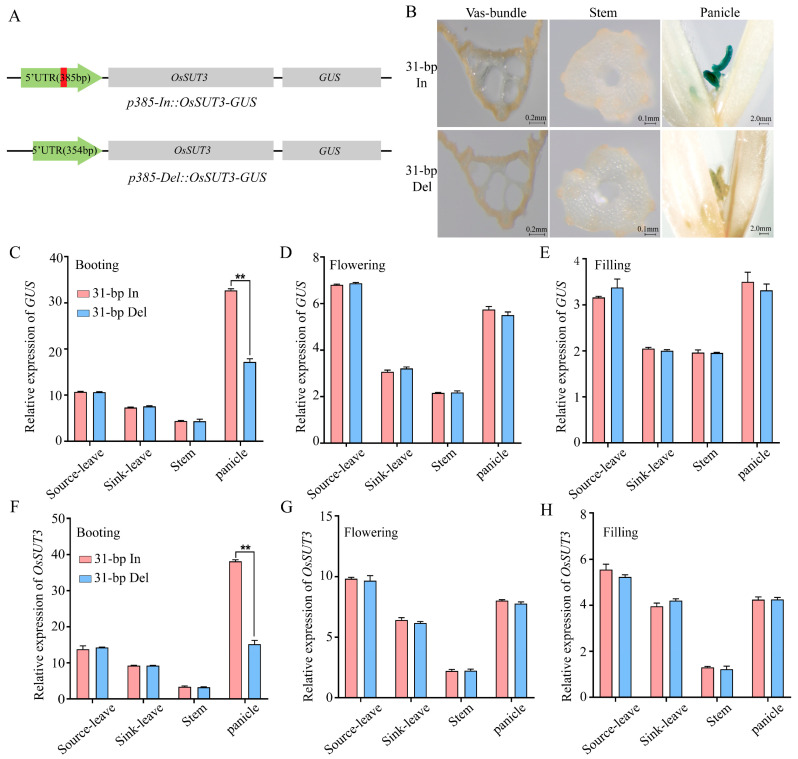
Regulation of 31-bp InDel on the expression of *OsSUT3* and *GUS*. (**A**): Linear diagrams of transgenic vectors of 31-bp In and 31-bp Del; note: the red box is 31-bp fragment. (**B**): GUS staining was used to detected the leaves, stems and panicle. (**C**–**E**): The relative expression of *GUS* gene of 31-bp In and 31-bp Del. (**F**–**H**): The relative expression of *OsSUT3* gene of 31-bp In and 31-bp Del (** *p* < 0.01, the significance of the difference was analyzed using *t*-test; vertical bars indicate the mean value ± standard deviation from three independent experiments).

In our previous study, a close relationship between the expression level of the *OsSUT3* gene and the 31-bp sequence located in 5′UTR of *OsSUT3* was found in various rice accessions [10]. Additionally, we also found that 5′UTR can be used as a promoter, p385, specifically mediated maximal *GUS* expression in pollen tissues [7]. In order to clarify the effect of the 31-bp InDel mutation on *OsSUT3* expression, we constructed two transgenic vectors, *p385-In::OsSUT3-GUS* and *p385-Del::OsSUT3-GUS* (Figure 2A), and obtained positive transgenic plants, 31-bp In and 31-bp Del, using an agrobacterium-mediated method.

The mRNA expression profiles of reporter gene *GUS* and downstream gene *OsSUT3* (Figure 2C–H) among different tissues (leave, stem and panicle) at booting, flowering, and filling stages were investigated firstly. The results show that the expression patterns of *GUS* and *OsSUT3* genes were very similar, and they both expressed widely in all tested tissues at three stages, and has the highest mRNA expression level in panicle at booting stage and lowest in stem at the filling stage (Figure 2C,E,F,H). In addition, the expressions of *GUS* and *OsSUT3* were generally high in panicle at three stages (Figure 2C–H). Additionally, the expressions of two genes in panicle of 31-bp In lines was far higher than that of 31-bp Del lines, *GUS* and *OsSUT3* expressions in panicle of 31-bp In were, respectively, 1.90 and 2.51 times higher than those of 31-bp Del (Figure 2C,F).

This study focuses on pollen development; thus, the histochemical staining of GUS protein in different tissues was explored at the booting stage. The results show that both in 31-bp In and 31-bp Del, the expression of *GUS* was the highest in panicle and was mainly concentrated in the anther (Figure 2B,C). Consistent with mRNA expression, it was found that very high GUS activity was induced in the anther of 31-bp In lines at booting stage.

### 2.3. Overview of the Transcriptomic Differences to 31-bp InDel

In order to understand the molecular mechanism of the 31-bp InDel fragment regulating the rice *OsSUT3* gene and affecting the sucrose transport process, we performed transcriptome sequencing on the panicles of 31-bp In and 31-bp Del at the booting stage. After eliminating the adaptor and poor-quality sequences, in total, 49,827,635 bp and 44,718,088 bp clean reads were retrieved from the 31-bp In and 31-bp Del samples, respectively. The average GC content varied from 48.54% to 48.68% in different libraries, and the Q30 percentage exceeded 92.50% (Table 1).

A total of 94,545,723 clean reads were generated by sequencing six cDNA libraries. All reads were classified into three categories: total mapped, multiple mapped and uniquely mapped. From the total clean read, 96.32–96.54% was totally mapped, 92.90–92.93% was uniquely mapped, and 3.38–3.63% was multiply mapped. Moreover, 47.96–48.03% was counts of reads mapped to the sense chain, and 48.03–48.12% was counts of reads mapped to the antisense chain (Table 2). In addition, more than 83.03% of the readings were mapped to the exon region of the reference genome (Figure S2).

**Figure 3:**
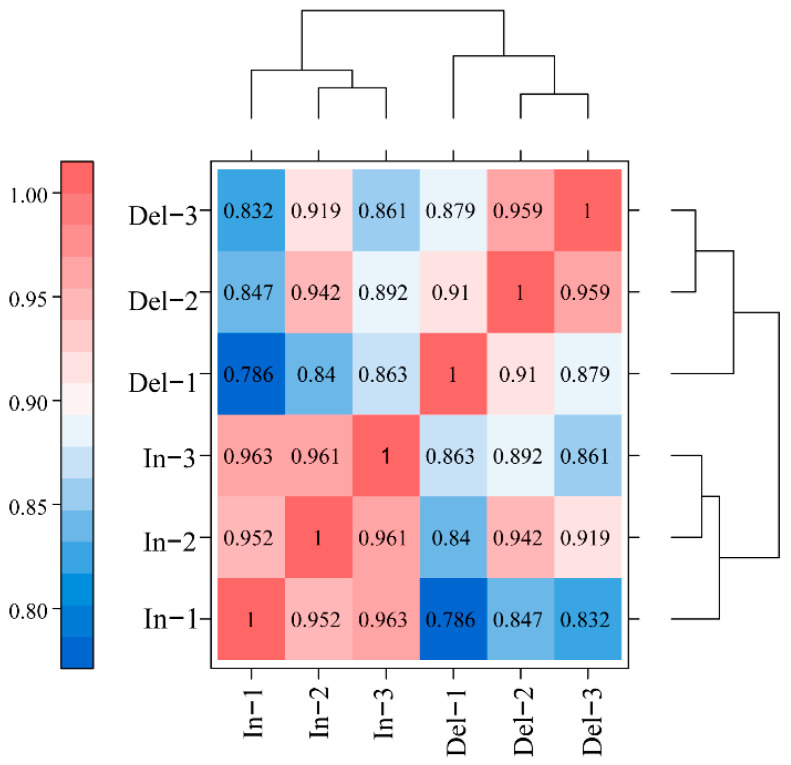
Heat-map diagram of inter-sample correlation analysis. Note: color blocks indicate the correlation coefficient values, the blue color indicates a lower correlation and the red color represents a higher correlation coefficient between samples.

Finally, the sequence and expression information of 39,036 genes were obtained for subsequent analysis. According to the FPKM density distribution comparison diagram of each sample, all biological replicates showed similar expression patterns, indicating that our sequencing data have high reliability (Figure S3). In the correlation coefficient heat map of 31-bp In and 31-bp Del (Figure 3), the average correlation coefficient of 31-bp In (In-1, In-2 and In-3) was 0.916, and that of 31-bp Del (Del-1, Del-2 and Del-3) was 0.959, indicated that the expression levels of these samples were similar. All these data proved that our RNA-Seq analysis is reliable and the selected samples are reasonable.

### 2.4. DEGs Identification and Enrichment Analysis

**Figure 4:**
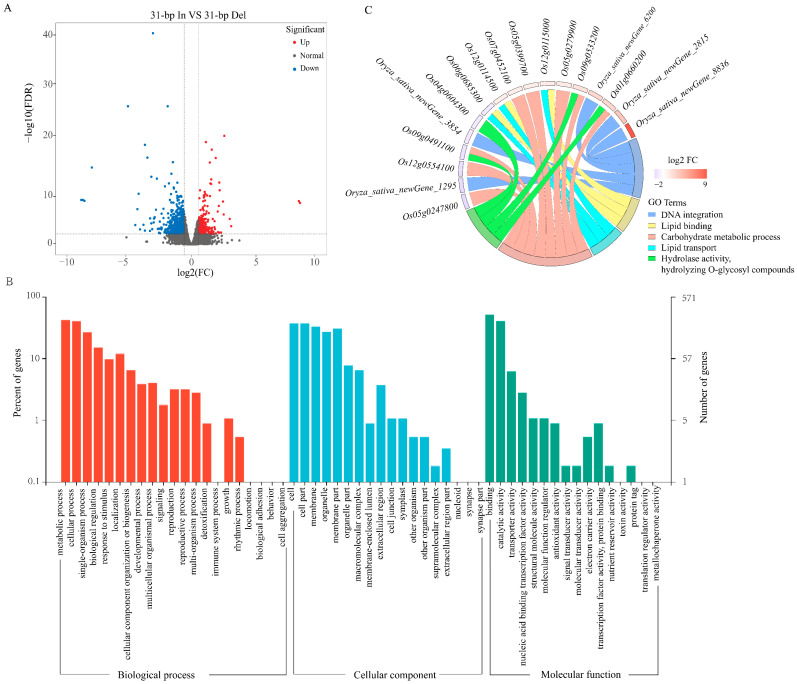
Distribution of DEGs and functional enrichment analysis. (**A**): Volcanic plot of DEGs between 31-bp In and 31-bp Del; note: *x*-axis: difference multiple values, *y*-axis: significance value. (**B**): GO enrichment analysis of DEGs. (**C**): Circos plot shows the main items and DEGs via GO analysis.

We systematically analyzed and identified differentially expressed genes (DEGs) between 31-bp In and 31-bp Del with the DESeq2 method, using fold change ≥ 1.5 and FDR < 0.01 as screening criteria. A total number of 1028 DEGs were identified; among them, 347 genes were up-regulated and 681 genes were down-regulated in 31-bp In Vs 31-bp Del (Figure 4A, Table S1).

A total of 1028 genes were allotted in gene ontology (GO) terms and only 571 (55.54%) DEGs were confirmed as annotated in the database, of which 234 genes were up- and 337 were down-regulated. To clarify their functionality, GOseq30 was used to classify DEGs into different functional categories. These genes were divided into 44 subclasses (Table S2). Among the DEGs, 37.14%, 40.18% and 22.68% were classified separately based on biological process, cellular component and molecular function (Figure 4B). The GO analysis showed that the DEGs were mostly involved in the biological process and cellular component. A total of 22 secondary items were significantly (*p* < 0.01) enriched in DEGs, in which the first 5 items were DNA integration (GO:0015074, gene ratio is 2.63%, the same below), lipid binding (GO:0008289, 2.10%), lipid transport (GO:0006869, 1.58%), carbohydrate metabolic process (GO:0005975, 3.85%), hydrolase activity, hydrolyzing O-glycosyl compounds (GO:0004553, 2.63%). Among them, ‘carbohydrate metabolic process’ was enriched in the largest number of DEGs, including 22 genes between 31-bp In and Del (Table S3). Further, *Os01g0660200* (*OsChib3a*), *Os12g0554100* (*OsTBL34*), *Os09g0533200*, *Os09g0491100* (*Os9bglu30*), *Os05g0399700* (*OsChia1d*), *Os05g0247800* (*OsXIP*) *Os05g0279900* (*OsPGL17*), and *Os07g0452100* were significantly (|log2FC| > 1) enriched in this item (Figure 4C).

### 2.5. Enriched Metabolic Pathways of DEGs by KEGG

**Figure 5:**
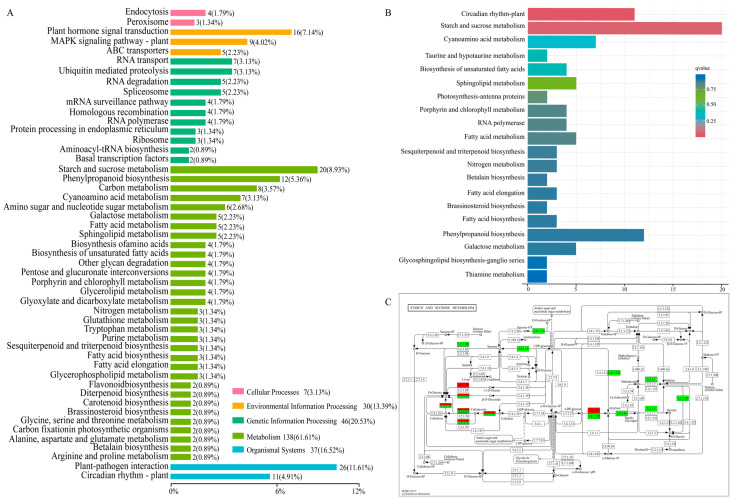
Enriched metabolic pathways of DEGs by KEGG. (**A**) KEGG classification on DEGs; note: *y*-axis: KEGG pathway terms; *x*-axis: Number and the percentage of genes annotated to the KEGG pathway. (**B**) TOP 20 pathways of KEGG enrichment; note: the q-value to determine the enrichment significance was calculated through hypergeometric distribution. (**C**) Starch and sucrose metabolic pathway in 31-bp Del. note: red indicates up-regulated genes, green indicates down-regulated genes.

Pathway enrichment analysis can help to further understand the biological function of genes. The Kyoto Encyclopedia of Genes and Genomes (KEGG) database was implemented to explore the pathways involved in the regulation of 31-bp insertion at the late pollen development stage. Bar-plot analysis in the KEGG database was used to identify the DEG positioning of metabolic pathways. A total of 258 out of 1028 DEGs were found to be involved in five pathways: cellular processes, environmental information processing, genetic information processing, metabolism, and organismal systems (Figure 5A). Among them, ‘metabolism’ and ‘genetic information processing’ were significantly changed including both up- and down-regulation in panicles of genetic plants with 31-bp insertion (Figure 5B). In addition, the top 10 KEGGs (64 DEGs) in bar-plot showed that the DEGs of the ‘circadian rhythm-plant’ (11 DEGs) and ‘starch and sucrose metabolism’ (19 DEGs) pathways accounted for nearly half, 17.19% and 29.69%, respectively. The largest number of DEGs was enriched in the ‘starch and sucrose metabolism’ pathway, and it was similar to that annotated in ‘carbohydrate metabolic process’ via GO analysis. Furthermore, we found that most of the DEG related to sucrose and starch synthesis were down-regulated in 31-bp Del. (Figure 5C).

Except the expression of downstream genes, pollen fertility and panicle traits of yield-related were significantly reduced in 31-bp Del plants (Figure 1). To investigate the underlying mechanism, we correlated DEGs with significantly changed sucrose (Table 3). *Os02g0744700* (starch synthase gene), *Os06g0194900* (sucrose phosphate synthase gene) and *Os02g0771700* (glycoside hydrolase gene) were highly correlated (|PCC| > 0.9) with sucrose content. We hypothesized that these 12 genes play important roles in regulating the sucrose and starch synthesis pathway of rice panicles under 31-bp InDel. Additionally, we found that two genes involved in sucrose synthesis, *Os01g0919400* (sucrose phosphate synthase gene) and *Os06g0194900* (sucrose synthase gene), and two genes involved in starch synthesis, *Os06g0133000* (granule-bound starch synthase gene) and *Os04g0409200* (starch branching enzyme gene), were positively correlated with an increase in sucrose content (|PCC| > 0.8). Furthermore, five genes related to glucose metabolism, *Os02g0733300* (glycoside hydrolase gene), *Os02g0771700* (glycoside hydrolase gene), *Os06g0675700* (alpha-glucosidases gene), *Os08g0114200* (glycoside hydrolase gene) and *Os01g0946500*, were highly correlated (|PCC| > 0.8) with sucrose and starch synthesis.

### 2.6. Expression Analysis of Sucrose and Starch Biosynthesis-Related Genes under 31-bp Deletion

**Figure 6:**
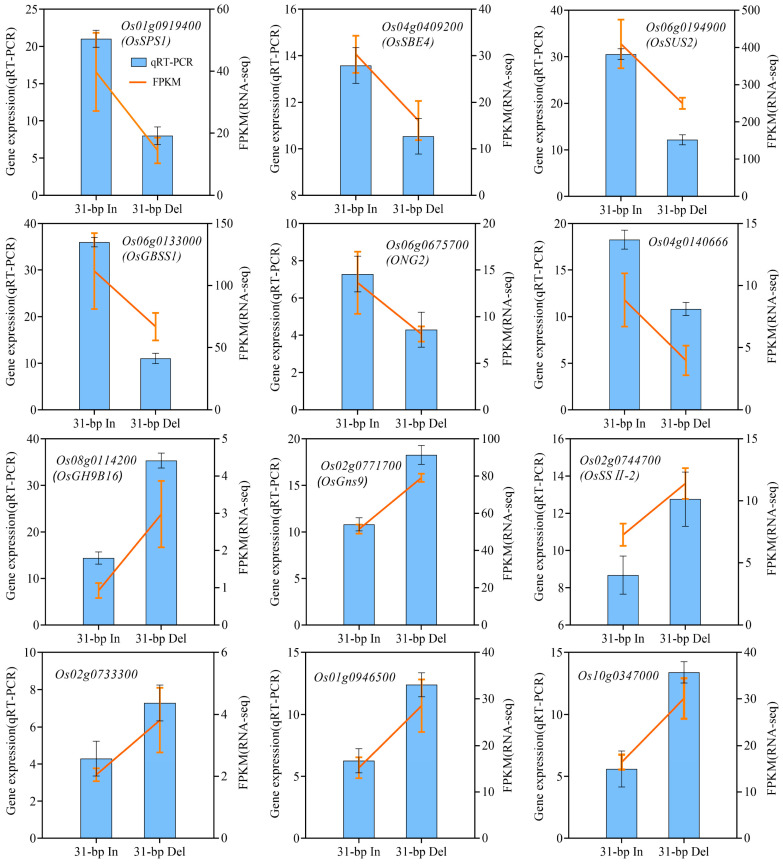
The expression trends of qRT-PCR and RNA-seq of top twelve sucrose-related DEGs. Note: histograms represent the results of the qRT-PCR assays, using the 2^−ΔΔCt^ algorithm, with the scale on the left ordinate of each graph. Orange lines represent the results of the FPKM analyses, with the scale on the right ordinate of each graph.

In total, the expressions of top twelve DEGs selected in the pathway of starch and sucrose metabolism were consistent with the transcriptome analysis results, which confirmed the accuracy of the RNA-Seq results (Figure 6).

**Figure 7:**
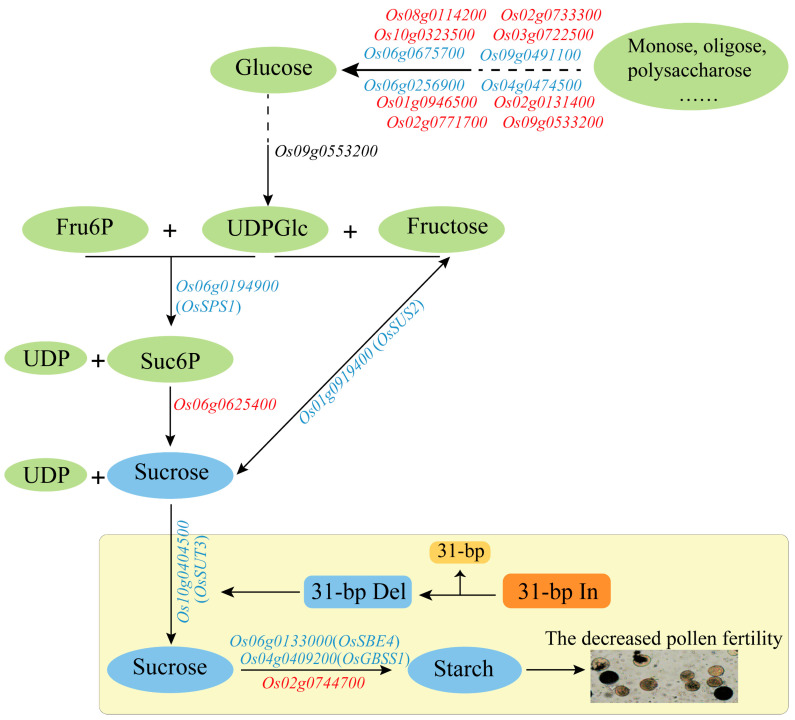
Pathway of 31-bp InDel regulating the formation of sucrose and starch in pollen. Note: red font indicates up-regulated genes, blue font indicates down-regulated genes.

According to the GO and KEGG results, ‘carbohydrate metabolic process’ and ‘starch and sucrose metabolism’ were significantly enriched. These findings may elucidate the regulated metabolism of 31-bp InDel to pollen development. 31-bp deletion directly affects the sucrose and starch accumulate and synthesis, causing the pollen fertility to decrease. In this study, we combined these two significant metabolic pathways associated with DEGs, and the mechanism underlying the formation of sucrose and starch in pollen has been mapped (Figure 7). Among DEGs, the genes of *Os10g0404500* (*OsSUT3*)—sucrose transporter protein—*Os06g0194900* (*OsSPS1*) and *Os06g0194900* (*OsSUS2*)—sucrose synthesis-related enzymes—and *Os04g0409200* (*OsSBE4*) and *Os06g0133000* (*OsGBSS1*)—starch synthesis-related enzymes—were all downregulated in 31-bp-deleted panicles. These genes might have vital roles in accumulating the sucrose and starch in panicle, especially in pollen, and could provide information on pollen starch biosynthesis mechanisms in rice.

## 3. Discussion

Crop genetics resources serve both as primary materials for genetic improvement and important safeguards for sustainable breeding. Furthermore, some genetic resources can also be used to explore the unclear molecular basis for the key agronomic traits found in some breeds. Pollen fertility in rice is a quantitative trait that is controlled by multiple genes. *OsSUT3,* as a member of the sucrose transporter in rice, may be a major role in pollen development [9,21,22,23]. A 31-bp InDel influencing the expression of downstream gene was identified in the 5′UTR of *OsSUT3* [10].

InDel variants within a non-coding region have been demonstrated to result in a modified phenotype in other studies [24,25]. Thus, an InDel sequence can regulate the transcriptional expression of downstream target gene by binding transcription factors or miRNA [26]. In our investigation of the 31-bp InDel effects on gene expression and main agronomic traits, we found that the *OsSUT3* and *GUS* expression in the panicle of 31-bp insertion plants at booting stage was significantly higher than that of 31-bp deletion plants (Figure 2C,F). In accordance with *OsSUT3* expression, the biggest difference in sucrose content between 31-bp insertion and deletion transgenic plants existed in the panicle at booting stage (Figure 1L). Except for that, pollen fertility, pollen viability and seed setting rate in 31-bp deletion plants decreased prominently (Figure 1H–K and Figure S1). A possible explanation is that the decreased expression of *OsSUT3* influenced the sucrose transport into rice pollen. Additionally, it directly leads to the deficiency of starch synthesis in pollen, thus reducing pollen fertility and seed setting rate [27,28,29]. Our data suggest that the 31-bp InDel in the 5′UTR of *OsSUT3* is effective as regards rice pollen development and yield. According to our previous study, the 31-bp polymorphism locus resulted in a positive selection pressure in rice domestication [10]. However, the mechanism through which this locus contributes to the transcriptional regulation of *OsSUT3* and the starch accumulation of rice pollen require further investigation.

In this study, we carried out a comprehensive RNA-seq experiment to study the transcriptional profiles of the panicles in 31-bp insertion and deletion rice transgenic plants at booting stage. The 31-bp InDel polymorphism is the major factor determining the transcription change in the same genetic background of Nipponbare. Our data showed that more DEGs with decreased expression (347 upregulated and 681 downregulated, 1028 in total) were identified in 31-bp deletion lines; thus, we concluded that the 31-bp sequence was a key transcription factor binding site for promoting gene expression. It has been demonstrated that InDel variants in a non-coding region, such as a promoter, 5′UTR, and intron, regulate gene expression and are closely associated with growth and reproductive traits in multiple animals [30,31,32]. Thus, it was essential to compare 31-bp insertion data with 31-bp deletion data to identify DEGs at the late stage of rice pollen development.

In our transcriptome analysis, we found that ‘starch and sucrose metabolism’ was the most significantly enriched pathway in the KEGG analysis, and ‘metabolic process’ and ‘binding’ were the most significantly enriched terms in the GO analysis (Figure 4B, Table S2). These results strongly indicate that the carbohydrate metabolism machinery was regulated when the 31-bp sequence was deleted. Consistent with our qRT-PCR results, the expressions of the downstream gene in transgenic plants without 31-bp showed significant decline (Figure 2C,F). The downstream gene *OsSUT3* encodes a sucrose transporter protein that affects pollen starch accumulation in rice [22]. Therefore, it is possible that the 31-bp region was demanded for the binding site during the high efficiency transcription of downstream gene.

The starch and sucrose metabolism pathway was enriched in the DEG group with 19 genes; therefore, these genes were considered the ‘core’ gene in 31-bp regulated transcriptional reprogramming. The expression of most of the core genes (12 DEGs) was verified via qRT-PCR, and the expression trend was consistent with the transcriptome (Figure 6). Among the core genes, the three genes with the most significant differences in expression, *OsGBSS1*, *OsSPS1*, and *OsSUS2*, have been functionally characterized as important regulators of starch synthesis in rice pollen and endosperm [33,34,35]. Thus, the core genes were likely common genes involved in starch synthesis and the accumulation of rice pollen. Supporting this conclusion are the disruption of *OsSPS1* results in sterile pollen as abnormally accumulating starch during pollen development [36], and the reduction in *OsSUS2* transcript levels will decrease the spikelet number by affecting sucrose decomposition and starch biosynthesis [35,37], and GBSS1 encoded by *Wx* gene is well-known to be positively correlated with amylose content (AC) in endosperm [38,39]. We speculate that the 31-bp sequence might be a binding site of transcription factor for inducing *OsSUT3* expression and regulating the sucrose metabolism in rice pollen.

In summary, the 31-bp InDel located in 5′UTR of *OsSUT3* significantly regulates the downstream genes in the panicle of the *p385-In/Del::OsSUT3-GUS* transgenic plant at the booting stage. Additionally, this study provides a high-quality, comprehensive RNA-seq dataset for expression regulation of the 31-bp InDel and enhances our understanding of the transcriptional networks in regulatory effect of the 31-bp sequence, highlighting possible candidate genes that may play important roles in rice pollen starch synthesis and fertility.

## 4. Materials and Methods

### 4.1. Plasmid Construction and Transformation

In this study, the promoter p385 is the 5′UTR region of the *OsSUT3* [7], and the 5′UTR region of the 31-bp insertion and 31-bp deletion types is 385 and 354 bases long, respectively. The promoter p385 was fused with the *OsSUT3* gene and the *GUS* reporter gene to construct the recombinant vectors *p385-In::OsSUT3-GUS* and *p385-Del::OsSUT3-GUS*. Subsequently, the recombinant constructs were mobilized into A. tumefaciens (strain GV3101) using a protocol described in previous studies [36].

### 4.2. Plant Materials and Growth Conditions

The seeds of *japonica* rice cultivar Nipponbare, obtained from the Rice Research Institute of Yunnan Agricultural University (Kunming, China), were disinfected with 0.1% HgCl_2_ solution, followed by washing with autoclaved water that was used for genetic transformation. Calli derived from the mature embryos were infected with an agrobacterium culture, and putatively transformed calli were selected on a Murashige and Skoog (MS) medium containing 100 mg/L kanamycin (Kan). Plants regenerated from the selected Kan-tolerant calli were grown to maturity in the greenhouse of the Rice Research Institute of Yunnan Agricultural University (Kunming, China).

### 4.3. GUS Staining

The leaves, stems and panicles of the rice transgenic plants were collected for a histochemical analysis 1 or 2 days pre-anthesis. All the explants were stained with a GUS stain kit (Cat. No. G3061; Solarbio Co.Ltd., Beijing, China) according to the instructions.

### 4.4. Total RNA Extraction and qRT-PCR Analysis of Gene Expression

The rice tissue samples, including source leaves (flag leaves), sink leaves (sheaths of the 3rd leaves from the top), stems and panicles at booting, flowering, and filling stages, to be tested (50–100 mg fresh weight) were ground into powder with liquid nitrogen and a mini pestle; then, the total RNA of the samples was extracted using a Trizol kit (Cat. No. DP424; Tiangen Co., Ltd., Beijing, China). Next, 1 μL of dNase was added to the RNA solution (30–50 µL) to remove DNA contamination. The concentration of RNA samples was estimated using a spectrophotometer (NanoDrop 1000; Thermo Scientific Inc., Waltham, MA, USA), and the quality of the RNA samples was examined using a 1.2% denatured agarose gel (Cat. No. 111860; XHLY Co., Ltd., Beijing, China).

The first-strand cDNA was synthesized via the addition of an equal quantity of RNA using a FastKing RT Kit (Cat. No. FP205-02; Tiangen Co.Ltd., Beijing, China) for qRT-PCR. Primers were designed for each gene using Primer 5 software (Table S4). The *β-actin* gene was used as an endogenous control. qRT-PCR was performed using a CFX96 Real Time System (Bio-Rad, Hercules Co., Ltd., New York, NY, USA) and a Super Real Premix Plus (SYBR Green) (Cat. No. FP205-01; Tiangen Co., Ltd., Beijing, China) according to the instructions. The relative quantification values were calculated using the 2^−ΔΔCt^ method [37], and three independent biological replicates were analyzed.

### 4.5. Detection of Pollen Fertility and Viability

Firstly, select three florets (anthers up to 2/3 of the spikelet hull) of an independent plant, and then take two anthers from each floret. Crush the anthers with tweezers and stain with I_2_-KI (1% *w*/*v*) solution or Alexander solution (Cat. No. G3050; Solarbio Co., Ltd., Beijing, China) for 5–10 min. Finally, place these under 80× optical microscope (Nikon SMZ1000, OLYMPUS, Tokyo, Japan) to observe the pollen staining. Three different visual fields are randomly selected for photo recording and the procedure is repeated five times foreach sample.

I_2_-KI solution was used to detect pollen fertility and Alexander solution was used to detect pollen viability.

### 4.6. Determination of Sucrose Content

The middle panicle tissues at booting (11 stages of pollen development [38]), flowering, and filling (10 days after flowering) stages to be tested were taken and operated according to the instructions of the sucrose content determination kit (Cat. No. G0506F, Grace Biotechnology Co., Ltd., Suzhou, China). The determination was performed under a spectrophotometer (uvmini1240, SHIMADZU, Kyoto, Japan) at a wavelength of 620 nm, and each sample was analyzed three times.

### 4.7. Determination of Panicle Character

The 20 panicles of the 31-bp In and Del transgenic plants at the maturity stage were respectively investigated for surveying the panicle-related agronomic traits, which including panicle length (cm), branch number, spikelet number per panicle, seed setting rate (%), 1000-grain weight (g), grain length (mm), grain width (mm) and grain thickness (mm). 

### 4.8. Library Preparation for Transcriptome Sequencing

The panicle RNA of 31-bp In and 31-bp Del at the booting stage was extracted, respectively. The RNA extraction method was the same as above.

A total amount of 1μg RNA per sample was used as the input material for the RNA sample preparations. Sequencing libraries were generated using NEBNext UltraTM RNA Library Prep Kit for Illumina (NEB, Ipswich, MA, USA) following the manufacturer’s recommendations, and index codes were added to attribute sequences to each sample. Briefly, mRNA was purified from total RNA using poly-T oligo-attached magnetic beads. Fragmentation was carried out using divalent cations under elevated temperature in NEBNext First Strand Synthesis Reaction Buffer (5×). First strand cDNA was synthesized using random hexamer primer and M-MuLV Reverse Transcriptase. Second strand cDNA synthesis was subsequently performed using DNA Polymerase I and rNase H; the remaining overhangs were converted into blunt ends via exonuclease/polymerase activities. After adenylation of 3′ ends of DNA fragments, NEBNext Adaptor with hairpin loop structure were ligated to prepare for hybridization. In order to preferentially select cDNA fragments of 240 bp in length, the library fragments were purified with AMPure XP system (Beckman Coulter, Beverly, CA, USA). Then, 3 µL USER Enzyme (NEB, MA, USA) was used with size-selected, adaptor-ligated cDNA at 37 °C for 15 min followed by 5 min at 95 °C before PCR. Then, PCR was performed with Phusion High-Fidelity DNA polymerase, Universal PCR primers and Index (X) Primer. At last, PCR products were purified (AMPure XP system) and the library’s quality was assessed on the Agilent Bioanalyzer 2100 system.

### 4.9. Data Filtering, Reads Mapping and RNA-Seq Data Analysis

Raw data (raw reads) of fastq format were firstly processed through in-house perl scripts. In this step, clean data (clean reads) were obtained by removing reads containing adapter, reads containing ploy-N and low-quality reads from raw data. At the same time, Q20, Q30, GC-content and sequence duplication level of the clean data were calculated. All the downstream analyses were based on clean data with high quality.

The adaptor sequences and low-quality sequence reads were removed from the data sets. Raw sequences were transformed into clean reads after data processing. These clean reads were then mapped to the rice reference genome sequence (*Oryza_sativa*. IRGSP-1.0. genome; https://rapdb.dna.affrc.go.jp/ accessed on 1 January 2022.). Only reads with a perfect match or one mismatch were further analyzed and annotated based on the reference genome. HISAT2 tools were used to map the reference genome [39].

### 4.10. Transcriptomic Analysis

Differential expression analysis of two groups was performed using the DESeq2. DESeq2 provide statistical routines for determining differential expression in digital gene expression data using a model based on the negative binomial distribution. The resulting *p* values were adjusted using the Benjamini and Hochberg’s approach for controlling the false discovery rate. Genes with an adjusted fold change ≥ 1.5 and FDR < 0.01 found via DESeq2 were described as differentially expressed.

Next, we performed gene ontology (GO) enrichment analysis of differentially expressed genes (DEGs) using the gOseq R program package based on Wallenius non-central hypergeometric distribution [40], and used KOBAS software to test the enrichment of DEGs in KEGG pathways [41]. GO and KEGG analyses were performed using BMKCloud (www.biocloud.net/, accessed on 1 December 2022.).

### 4.11. Statistical Analysis

The original data were compiled using MS Excel 2019. All primers were designed using Primer Premier 5 software. All data were analyzed using IBM SPSS Statistics 26. All graphics were built using GraphPad Prism 8.0 and Adobe Illustrator 2021.

## Figures and Tables

**Table 1:** Summary of reads of the samples retrieved from Illumina sequencing.

Samples	Clean Reads	Clean Bases	Q30 (%)	GC Content (%)
31-bp In	49,827,635	7,437,804,261	92.72	48.68
31-bp Del	44,718,088	6,667,648,177	92.50	48.54

**Table 2:** Statistical summary of alignment results of RNA-seq used in mapping.

Samples	Total Clean Reads	Clean Read Mapped (%)	Uniquely Mapped (%)	Multiple Mapped (%)	Reads Map to Sense Chain (%)	Reads Map to Antisense Chain (%)
31-bp In	48,100,141	96.54	92.90	3.63	48.03	48.12
31-bp Del	43,067,200	96.32	92.93	3.38	47.96	48.03

**Table 3:** Key DEGs related to sucrose.

Pathway	Gene ID	Gene Description	In VS Del	PCC
Starch and sucrose metabolism	*Os01g0919400*	Sucrose phosphate synthase, sucrose synthesis in pollen germination	down	0.865
	*Os04g0140666*	Similar to OSIGBa0124C14.6 protein	down	0.849
	*Os04g0409200*	Starch branching enzyme IIa, starch biosynthesis	down	0.890
	*Os06g0133000*	Granule-bound starch synthase, synthesis of amylose in endosperm	down	0.815
	*Os06g0194900*	Sucrose synthase 2	down	0.912
	*Os06g0675700*	Alpha-glucosidase, α-glucosidase in rice seeds	down	0.831
	*Os08g0114200*	Glycoside hydrolase family 9 subclass B16	up	−0.855
	*Os10g0347000*	X8 domain containing protein	up	−0.892
	*Os01g0946500*	Similar to Glucan endo-1,3-beta- glucosidase GV	up	−0.881
	*Os02g0733300*	Glycoside hydrolase family 9 subclass B1	up	−0.865
	*Os02g0744700*	Similar to starch synthase isoform zSTSII-2	up	−0.934
	*Os02g0771700*	Glycoside hydrolase, family 17 protein	up	−0.990

Note: The PCC value is calculated via the Pearson correlation method.

## Data Availability

Not applicable.

## Supplementary Materials

The following supporting information can be downloaded at: https://www.mdpi.com/article/10.3390/ijms241310640/s1.

## References

[1] Tran T.M., Hampton C.S., Brossard T.W., Harmata M., Robertson J.D., Jurisson S.S., Braun D.M. (2017). In vivo transport of three radioactive [18F]-fluorinated deoxysucrose analogs by the maize sucrose transporter ZmSUT1. Plant Physiol. Bioch..

[2] Kühn C., Grof C.P. (2010). Sucrose transporters of higher plants. Curr. Opin. Plant Biol..

[3] Hu Z., Tang Z., Zhang Y., Niu L., Yang F., Zhang D., Hu Y. (2021). Rice SUT and SWEET transporters. Int. J. Mol. Sci..

[4] Aoki N., Hirose T., Scofield G.N., Whitfeld P.R., Furbank R.T. (2003). The sucrose transporter gene family in rice. Plant Cell Physiol..

[5] Hirose T., Zhang Z., Miyao A., Hirochika H., Ohsugi R., Terao T. (2010). Disruption of a gene for rice sucrose transporter, OsSUT1, impairs pollen function but pollen maturation is unaffected. J. Exp. Bot..

[6] Takeda T., Toyofuku K., Sobolewska A., Matsukura C., Yamaguchi J. (2001). Sugar transporters involved in flowering and grain development of rice. J. Plant Physlol..

[7] Li D.D., Xu R.C., Lv D., Zhang C.L., Yang H., Zhang J.B., Wen J.C., Li C.Y., Tan X.L. (2020). Identification of the core pollen-specific regulation in the rice *OsSUT3* promoter. Int. J. Mol. Sci..

[8] Hinnebusch A.G., Ivanov I.P., Sonenberg N. (2016). Translational control by 5’-untranslated regions of eukaryotic mRNAs. Science.

[9] Chen C.H., Lin H.Y., Pan C.L., Chen F.C. (2011). The plausible reason why the length of 5' untranslated region is unrelated to organismal complexity. BMC Res. Notes..

[10] Wang N., Cheng M., Chen Y., Liu B.J., Wang X.N., Li G.J., Zhou Y.H., Luo P., Xi Z.Y., Yong H.J. (2021). Natural variations in the non-coding region of ZmNAC080308 contributes maintaining grain yield under drought stress in maize. BMC Plant Biol..

[11] Feng Y.M., Liu M., Wang Z., Zhao X.L., Han B., Xing Y.P., Wang M.Y., Yang Y. (2019). A 4-bp deletion in the 5’UTR of *TaAFP-B* is associated with seed dormancy in common wheat (*Triticum aestivum* L.). BMC Plant Biol..

[12] Tong J.P., Han Z.S., Han A.N., Liu X.J., Zhang S.Y., Fu B.Y., Hu J., Su J.P., Li S.Q., Wang S.J. (2016). *Sdt97*: A point mutation in the 5’untranslated region confers semidwarfism in rice. G3-Genes Genom. Genet..

[13] Zhang C.L., Li Q.P., Yang H., Wang T., Li J., Wen J.C., Jin S.L., Zhang Z.L., Chen L.J., Li D.D. (2022). A 31-bp indel localised in the 5’untranslated region of *OsSUT3* affects the gene expression and rice (*Oryza sativa* L.) pollen development. Czech J. Genet. Plant Breed..

[14] Hazen S.P., Wu Y.J., Kreps J.A. (2003). Gene expression profiling of plant responses to abiotic stress. Funct. Integr. Genomics.

[15] Oh E., Zhu J.Y., Wang Z.Y. (2012). Interaction between BZR1 and PIF4 integrates brassinosteroid and environmental responses. Nat. Cell Biol..

[16] Luo M.M., Liu X.X., Su H.Y., Li M.L., Li M.F., Wei J.H. (2022). Regulatory networks of flowering genes in angelica sinensis during vernalization. Plants.

[17] Wu Y.F., Lee S.K., Yoo Y., Wei J.H., Kwon S.Y., Lee S.W., Jeon J.S., An G. (2018). Rice transcription factor OsDOF11 modulates sugar transport by promoting expression of sucrose transporter and SWEET genes. Mol. Plant..

[18] Lee S.K., Lee J., Jo M., Jeon J.S. (2022). exploration of sugar and starch metabolic pathway crucial for pollen fertility in rice. Int. J. Mol. Sci..

[19] Li J.B., Kim Y.J., Zhang D.B. (2022). Source-to-sink transport of sugar and its role in male reproductive development. Genes.

[20] Jansing J., Buyel J.F. (2019). The correlation between DsRed mRNA levels and transient dsred protein expression in plants depends on leaf age and the 5’Untranslated region. Biotechnol. J..

[21] Chen W.C., Yang G.P., He Y., Zhang S.M., Chen H.Y., Shen P., Chen X.D., Huang Y.P. (2015). Nucleotides flanking the start codon in hsp70 mRNAs with very short 5’UTRs greatly affect gene expression in haloarchaea. PLoS ONE.

[22] Kim Y., Lee G., Jeon E., Sohn E.J., Lee Y., Kang H., Lee D.W., Kim D.H., Hwang I. (2014). The immediate upstream region of the 5’UTR from the AUG start codon has a pronounced effect on the translational efficiency in *Arabidopsis thaliana*. Nucleic Acids Res..

[23] Datta R., Chamusco K.C., Chourey P.S. (2002). Starch biosynthesis during pollen maturation is associated with altered patterns of gene expression in maize. Plant Physiol..

[24] Mu H., Ke J.H., Liu W., Zhuang C.X., Yip W.K. (2009). UDP-glucose pyrophosphorylase2 (*OsUgp2*), a pollen-preferential gene in rice, plays a critical role in starch accumulation during pollen maturation. Chin. Sci. Bull..

[25] Huang Z.Y., Gan Z.S., He Y.S., Li Y.H., Liu X.D., Mu H. (2011). Functional analysis of a rice late pollen-abundant UDP-glucose pyrophosphorylase (*OsUgp2*) promoter. Mol. Biol. Rep..

[26] Brenig B., Duan Y.Y., Xing Y.Y., Ding N.S., Huang L.S., Schütz E. (2015). Porcine *SOX9* gene expression is influenced by an 18 bp Indel in the 5’-Untranslated region. PLoS ONE.

[27] Simsek D., Tiu G.C., Flynn R.A., Byeon G.W., Leppek K., Xu A.F., Chang H.Y., Barna M. (2017). The mammalian ribo-interactome reveals ribosome functional diversity and heterogeneity. Cell.

[28] Ren T.H., Yang Y., Lin W.J., Li W.Y., Xian M.J., Fu R., Zhang Z.H., Mo G.D., Luo W., Zhang X.Q. (2020). A 31-bp indel in the 5’UTR region of *GNB1L* is significantly associated with chicken body weight and carcass traits. BMC Genet..

[29] Seung D. (2020). Amylose in starch: Towards an understanding of biosynthesis, structure and function. New Phytol..

[30] Chávez-Bárcenas A.T., Valdez-Alarcón J.J., Martínez-Trujillo M., Chen L., Xoconostle-Cázares B., Lucas W.J., Herrera-Estrella L. (2000). Tissue-specific and developmental pattern of expression of the rice *sps1* gene. Plant Physiol..

[31] Fan C.F., Wang G.Y., Wang Y.M., Zhang R., Wang Y.T., Feng S.Q., Luo K.M., Peng L.C. (2019). Sucrose synthase enhances hull size and grain weight by regulating cell division and starch accumulation in transgenic rice. Int. J. Mol. Sci..

[32] Hirose T., Hashida Y., Aoki N., Okamura M., Yonekura M., Ohto C., Terao T., Ohsugi R. (2014). Analysis of gene-disruption mutants of a sucrose phosphate synthase gene in rice, *OsSPS1*, shows the importance of sucrose synthesis in pollen germination. Plant Sci..

[33] Murata T., Sugiyama T., Minamikawa T., Akazawa T. (1966). Enzymic mechanism of starch synthesis in ripening rice grains. 3. Mechanism of the sucrose-starch conversion. Arch. Biochem. Biophys..

[34] Shapter F.M., Eggler P., Lee L.S., Henry R.J. (2009). Variation in Granule Bound Starch Synthase I (GBSSI) loci amongst Australian wild cereal relatives (Poaceae). J. Cereal Sci..

[35] Sano Y. (1984). Differential regulation of waxy gene expression in rice endosperm. Theor. Appl. Genet..

[36] Saha D., Kumar V., Bhat S.R., Srinivasan R. (2011). Upstream sequences of the *LOJ* gene leads to identification of a novel enhancer element conferring lateral organ junction-specific expression in *Arabidopsis thaliana*. Plant Mol. Biol. Rep..

[37] Arocho A., Chen B.Y., Ladanyi M., Pan Q.L. (2006). Validation of the 2^−ΔΔCt^ calculation as an alternate method of data analysis for quantitative PCR of BCR-ABL P210 transcripts. Diagn. Mol. Pathol..

[38] Zhang D.B., Luo X., Zhu L. (2011). Cytological analysis and genetic control of rice anther development. J. Genet. Genomics.

[39] Kim D., Langmead B., Salzberg S.L. (2015). HISAT: A fast spliced aligner with low memory requirements. Nat. Methods.

[40] Young M.D., Wakefield M.J., Smyth G.K., Oshlack A. (2010). Gene ontology analysis for RNA-seq: Accounting for selection bias. Genome Biol..

[41] Mao X.Z., Cai T., Olyarchuk J.G., Wei L.P. (2005). Automated genome annotation and pathway identification using the KEGG Orthology (KO) as a controlled vocabulary. Bioinformatics.

