# Supplementary material for: Transcriptome Analyses Show Changes in Gene Expression Triggered by a 31-bp InDel within OsSUT3 5′UTR in Rice Panicle
Source: Int J Mol Sci. 2023 Jun 26;24(13):10640. doi: 10.3390/ijms241310640 (PMC10341534; doi:10.3390/ijms241310640)
Supplement: Supplementary file 1 [file ijms-24-10640-s001.zip › Figure S1, S2, S3.pdf]

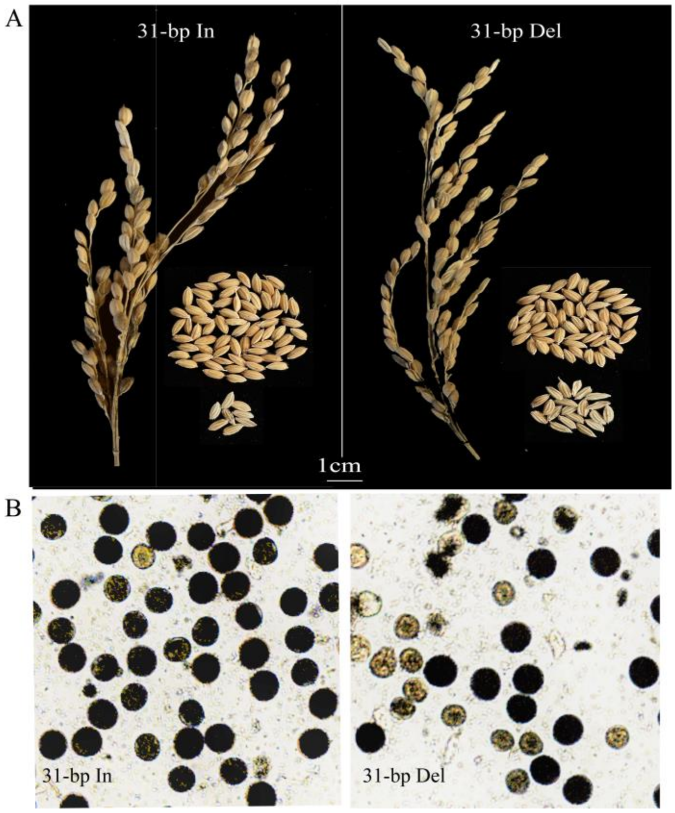

Figure S1. Panicle t and pollen of 31bp-In and 31bp-Del. (**A**): spikelet and grains of single panicle at mature stage (above, solid grains; below, empty grains). (**B**): I_2-KI staining of pollen grains.

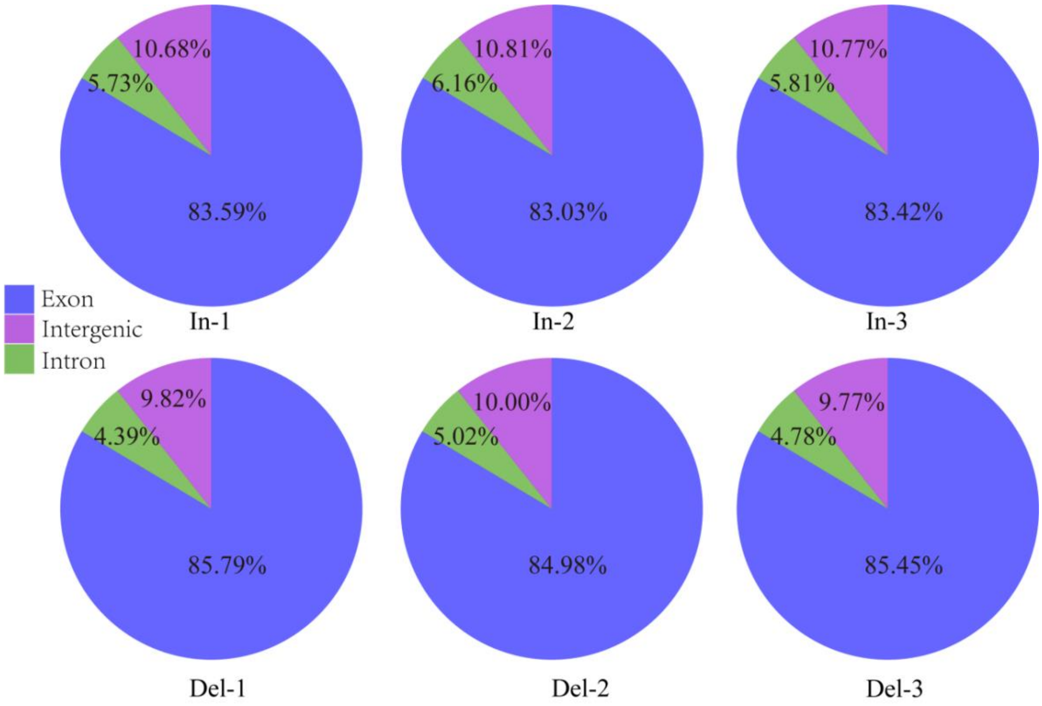

Figure S2. Reads distribution map of different regions of the genome. Note: The genome is divided into exon region, intergenic region and intron region. The region size is based on the percentage of Reads matched to the corresponding region in all Mapped Reads.

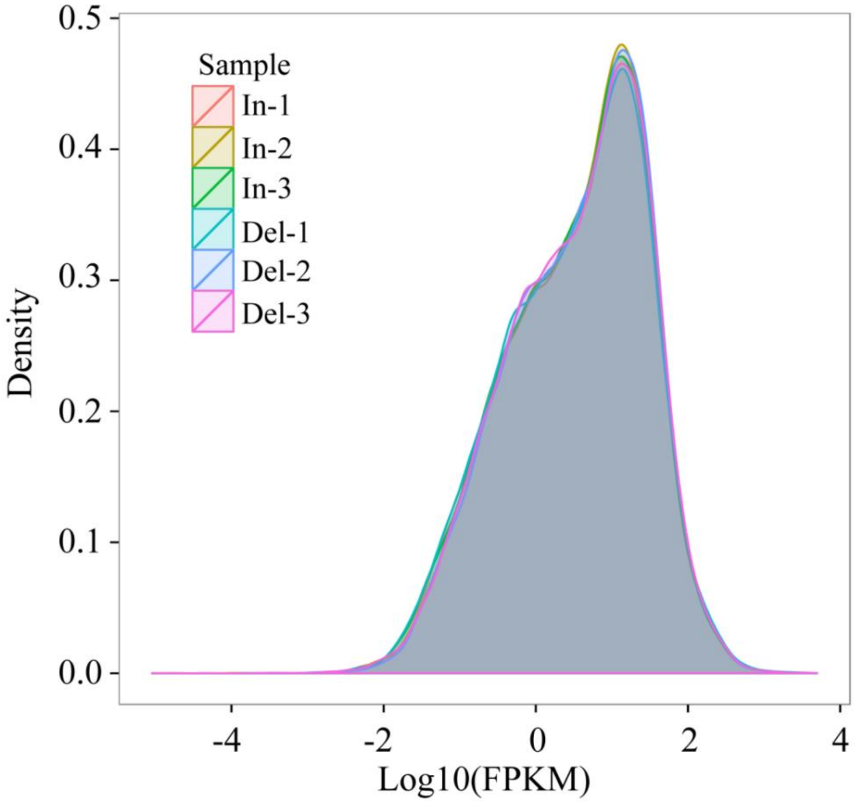

Figure S3. Comparison of FPKM density distribution of each sample. Note: The abscissa represents the logarithm of the corresponding sample FPKM, and the ordinate represents the probability density.
